# Burosumab and Dental Abscesses in Children With X‐Linked Hypophosphatemia

**DOI:** 10.1002/jbm4.10672

**Published:** 2022-09-20

**Authors:** Margaux Gadion, Agathe Hervé, Julia Herrou, Anya Rothenbuhler, Violaine Smail‐Faugeron, Frédéric Courson, Agnès Linglart, Catherine Chaussain, Martin Biosse Duplan

**Affiliations:** ^1^ Université Paris Cité Faculté de Santé (Unité de formation et de recherche Odontologie et Unité de formation et de recherche de médecine) Paris France; ^2^ Assistance publique des hopitaux de Paris, Reference Center for Rare Diseases of the Calcium and Phosphate Metabolism, OSCAR Network, Fédération hospitalo‐universitaire DDS‐net, European reference network BOND, Department of Dental Medicine Bretonneau Hospital Paris France; ^3^ Assistance publique des hopitaux de Paris, Reference Center for Rare Diseases of the Calcium and Phosphate Metabolism, OSCAR Network, European reference network BOND, Department of Rheumatology Cochin Hospital Paris France; ^4^ Assistance publique des hopitaux de Paris, Reference Center for Rare Diseases of the Calcium and Phosphate Metabolism, OSCAR Network, Endo‐European reference network and European reference network BOND, Department of Endocrinology and Diabetology for Children Bicêtre Paris Saclay Hospital Le Kremlin Bicêtre France; ^5^ Université Paris Saclay Institut national de la santé et de la recherche médicale unité mixte de recherche 1185 Physiologie et physiopathologie endocrinienne Le Kremlin Bicêtre France; ^6^ Université Paris Cité Laboratory Unité de recherche2496 Orofacial Pathologies, Imaging and Biotherapies Paris France; ^7^ Institut Imagine, INSERM U1163 Paris France

**Keywords:** DENTAL BIOLOGY, |MATRIX MINERALIZATION, BONE MATRIX, DISORDERS OF CALCIUM/PHOSPHATE METABOLISM, PTH/VIT D/FGF23, CELL/TISSUE SIGNALING – ENDOCRINE PATHWAYS

## Abstract

X‐linked hypophosphatemia (XLH) is a rare genetic disorder that disrupts skeletal and dental mineralization. In addition to rickets in children, XLH patients also have frequent spontaneous dental abscesses that increase the risk of tooth loss and may lead to facial cellulitis. Hypomineralized and hypoplastic dentin is the main driver of these infections. Conventional treatment (CT) of XLH improves this tissue defect and reduces the occurrence of dental abscesses. Burosumab is a recent treatment for XLH that targets excess circulating fibroblast growth factor 23 (FGF23), and its benefits on rickets have been demonstrated. It is not yet known whether burosumab improves dental manifestations of XLH. The main objective of our study was to compare the incidence of dental abscesses with XLH treated with either CT or burosumab. In this monocentric retrospective study, we measured and compared the incidence of dental abscess in children with XLH treated with either CT or burosumab, followed at our dental center for at least 1 year. The primary endpoint was the number of dental abscesses per month of dental follow‐up. A total of 71 children were included in the study, with a mean ± standard deviation (SD) age at the start of dental follow‐up of 7.86 ± 3.76. Thirty‐eight children were treated with CT (53.5%) and 33 with burosumab (46.5%). All children treated with burosumab had previously been treated with CT. The mean number of dental abscesses per month of dental follow‐up was significantly reduced in the burosumab group compared with the CT group (0.01 versus 0.04; *p* = 0.04). Burosumab treatment appears to be associated with a reduction in the number of dental abscesses in XLH children, compared with CT. © 2022 The Authors. *JBMR Plus* published by Wiley Periodicals LLC on behalf of American Society for Bone and Mineral Research.

## Graphical abstract

In a monocentric retrospective study, dental abscesses, a common manifestation of X‐Linked Hypophosphatemia, were less frequent in children treated with burosumab compared to those supplemented with phosphorus and vitamin D.

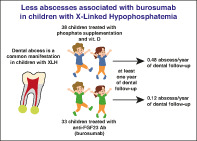

## Introduction

X‐linked hypophosphatemia (XLH) is a rare genetic disease that disturbs the mineralization of the skeleton and teeth.^(^ ^1^ ^)^ It affects approximately one in 20,000 births. In this disease caused by loss‐of‐function mutations in the gene encoding the enzyme phosphate regulating endopeptidase homolog X‐linked (PHEX), an excess of circulating fibroblast growth factor 23 (FGF23) acts on kidney tubules by increasing urinary phosphate excretion and disrupting vitamin D metabolism.^(^ ^2^ ^)^ In children, XLH manifests clinically by rickets responsible for growth retardation and limb deformities.^(^ ^3^ ^)^ In adults, XLH leads to osteoarthritis and osteomalacia responsible for fractures and delayed bone consolidation.^(^ ^3^, ^4^ ^)^ XLH patients also present with dental features, the main manifestations being spontaneous infections of endodontic origin (especially in children) and periodontitis (in adults).^(^ ^5^, ^6^ ^)^ These infections are very frequent,^(^ ^7^, ^8^, ^9^, ^10^ ^)^ increase the risk of tooth loss, and alter the quality of life of XLH patients.^(^ ^11^, ^12^ ^)^ The origin of these infections is the combination of hypomineralized and hypoplastic dentin,^(^ ^13^, ^14^ ^)^ enlarged pulp chambers with prominent pulp horns, and microcracks in the enamel, which favor the infection of the pulp chamber by oral bacteria. In children, these infections lead to dental abscesses, which translate clinically into painful swelling of the gum. If left untreated, these infections can lead to facial cellulitis, which are life‐threatening infections often requiring hospitalization.^(^ ^15^ ^)^

Conventional treatment (CT) of XLH consists of supplementation with phosphorus and active vitamin D analogues during growth. This treatment, when started early during growth and correctly followed by the patient, prevents or limits limb deformities and short stature.^(^ ^1^, ^16^ ^)^ CT also improves dentin formation and mineralization during tooth formation.^(^ ^17^ ^)^ This translates into a reduced prevalence of dental abscesses and reduced premature tooth loss in XLH patients treated with CT.^(^ ^18^, ^19^, ^20^ ^)^

In recent years, a new treatment for XLH, burosumab, a monoclonal antibody that targets excess circulating FGF23, has been developed for children^(^ ^21^ ^)^ and adults.^(^ ^22^ ^)^ This new treatment has demonstrated its value in restoring normal phosphatemia and reducing rickets severity in children.^(^ ^21^, ^23^ ^)^ In the *Hyp* mouse, a mouse model recapitulating XLH, a partial correction of dentin mineralization defects was observed with FGF23‐blocking antibody, notably by a reduction in the thickness of the pre‐dentin.^(^ ^24^ ^)^ However, the impact of burosumab treatment on dentin mineralization and the resulting occurrence of endodontic infections in XLH patients remains unknown, so far.

The primary objective of our research was to compare the incidence of dental abscesses in children and adolescents with XLH treated with either CT or burosumab. The secondary objective was to compare the incidence of cellulitis of odontogenic origin.

## Patients and Methods

### Study population

This study was retrospective and monocentric and followed Strengthening the Reporting of Observational Studies in Epidemiology (STROBE) guidelines. We included all individuals <18 years old diagnosed with XLH, referred to and followed in our center for oral health care from 2019 to 2022. The dental follow‐up period had to be at least 1 year in our center while taking the same treatment for XLH. Patients were excluded from the study if another general disease that may affect oral health was present, if information on the general treatment of XLH was missing, or if the period of dental follow‐up in our center was less than 1 year with the same treatment for XLH.

Patients received the conventional therapy with vitamin D analogues and phosphate supplements, according to international guidelines.^(^ ^1^ ^)^ Before starting burosumab, conventional therapy was stopped for 1 week. The dose of burosumab was started at 0.4 mg/kg and adjusted (increased or decreased as appropriate) according to fasting serum phosphate (sP). The injections of burosumab were performed every 2 weeks subcutaneously. According to French health authorities’ recommendations, the treatment goal was to reach sP >1.2 mmol/L (>3.7 mg/dL). The dose of burosumab never exceeded 2.0 mg/kg or 90 mg, even if this goal was not attained.

In the patients’ medical files, we collected and collated the following data: gender, age at onset of XLH treatment (in year), type of XLH treatment (conventional or burosumab), duration of the XLH treatment (month), age at onset of dental follow up in our center (year), duration of the dental follow‐up (month), alkaline phosphatase (ALP) levels during the dental follow‐up (IU/L), number of dental abscesses during the dental follow‐up period, number of cellulites of odontogenic origin during the follow‐up period, and dental caries during the follow‐up period. Patients were seen every 6 months with possible additional visits in case of emergency. Dental orthopantomogram were performed at the onset of dental follow‐up and repeated based on clinical needs.^(^ ^1^ ^)^ Dental abscesses were diagnosed based on clinical and radiological manifestations by dentists with experience of the oral complications of XLH (signs included pain, swelling of the gum or mucosa, fistula, and periapical radiolucency). Facial cellulites of odontogenic origin were diagnosed clinically and radiographically.

We calculated for each patient the number of dental abscesses and cellulites per month of dental follow‐up under the same XLH treatment as well as the mean ALP level during the dental follow‐up. Only dental abscesses and cellulitis diagnosed during the dental follow‐up period in our center were considered and counted.

When two dental orthopantomograms more than 1 year apart were available for a child, we compared the size of first molars pulp chambers on the two X‐rays.

### Statistics

Descriptive statistical analysis of the population and results were summarized as mean and standard deviation (SD) for continuous data and as *n* (%) for categorical data. The characteristics of participants according to the conventional treatment (CT) or burosumab treatment were assessed by bivariate analysis. The primary endpoint was the number of dental abscesses per month of dental follow‐up. A secondary endpoint was the number of cellulites of odontogenic origin per month of dental follow‐up. The association between the number of dental abscesses per month and the treatment received (burosumab or CT) was assessed using a linear regression model. Then variables significantly associated with the number of dental abscesses in univariate analysis were included in the multivariate linear regression model.

The subgroup comparison of the number of abscesses in patients who had had CT and then burosumab treatment was performed using a bivariate analysis for matched data.

For all the analyses, a *p* value <0.05 was considered significant. All analyses were performed on the statistical software R, version 3.5.2 (R Foundation for Statistical Computing, Vienna, Austria; https://www.r-project.org/).

### Regulatory approval

This study and protocols were designed, conducted, recorded, and reported in accordance with the principles established by the World Medical Association Declaration of Helsinki Ethical Principles for Medical Research Involving Human Subjects and local legislation and ethics guidelines. All participants were verbally informed about the potential use of their anonymized medical data for research purposes and the participants' non‐objection was collected. All participants had the right to withdraw from the study at any time by completing the form available from a link at http://recherche.aphp.fr/eds/droit-opposition. According to French law (*loi Jardé*), anonymous monocentric retrospective studies do not require institutional review board approval. However, the study was approved by the ethical committee of our institution (AP‐HP; approval provided on request). It was also approved by the French National Data Processing and Liberties Commission (CNIL).

## Results

The medical records of 99 children with XLH followed at our center were analyzed, of which 71 were included. Reasons for noninclusion were either a dental follow‐up shorter than 1 year (*n* = 26), the absence of any date for the onset of XLH treatment (*n* = 1), or a discontinued burosumab treatment (*n* = 1). The study sample consisted of 71 children, of whom 30 were boys (42.3%) and 41 were girls (57.7%). Thirty‐eight children were treated with the conventional treatment (CT) (53.5%) and 33 with burosumab (46.5%). Because the burosumab access was granted in 2018 and one indication for burosumab is the treatment of children who do not respond well to CT, all children in the burosumab group had been initially treated with CT.

In our sample, 19 children could be included in both groups; ie, they had at least 1 year of dental follow‐up on both treatments. To avoid including a child twice and to obtain groups of similar size, we decided to attribute these children to the burosumab group and not to the CT group. Of note, the comparison of the data for these 19 children when treated with CT and not included in the CT group and those of the 38 children treated with CT included in the sample did not show statistical differences for all parameters (Table S1).

Table 1 presents the characteristics of the samples. The mean ± SD age at XLH treatment onset was 2.92 ± 3.0 years in the CT group and 8.36 ± 3.81 in the burosumab group (*p* < 0.0001). The mean duration of XLH treatment was 97.9 ± 46.9 months in the CT group and 38.9 ± 29.0 in the burosumab group (*p* < 0.001). Mean ALP level was 385.8 ± 147.7 IU/L in the CT group and 339.8 ± 125.7 IU/L in the burosumab group. The mean age at dental follow‐up onset was 6.58 ± 3.33 years in the CT group and 9.33 ± 3.72 years in the burosumab group (*p* < 0.01). The mean duration of dental follow‐up was 53.9 ± 31.9 months in the CT group and 27.2 ± 9.6 months in the burosumab group (*p* < 0.001).

**Table 1:** Selected Participant Characteristics by Treatment

Variables	All patients (*n* = 71)	Conventional treatment (*n* = 38)	Burosumab treatment (*n* = 33)	Comparison between conventional treatment and burosumab (*p*)
Sex, *n* (%)				
Male	30 (42.3)	16 (42.1)	14 (42.4)	
Female	41 (57.7)	22 (57.9)	19 (57.6)	0.98
Age at XLH treatment onset (years), mean ± SD	5.45 ± 4.34	2.92 ± 3.0	8.36 ± 3.81	0.00001
XLH treatment duration (months), mean ± SD	70.5 ± 49.2	97.9 ± 46.9	38.9 ± 29.0	0.0001
Alkaline phosphatase level (IU/L), mean ± SD	364.4 ± 138.9	385.8 ± 147.7	339.8 ± 125.7	0.17
Age at dental follow‐up onset (years), mean ± SD	7.86 ± 3.76	6.58 ± 3.33	9.33 ± 3.72	0.004
Dental follow‐up duration (months), mean ± SD	41.5 ± 27.5	53.9 ± 31.9	27.2 ± 9.6	0.0009
Number of dental abscess per month of dental follow‐up (*n*/month), mean ± SD	0.03 ± 0.04	0.04 ± 0.05	0.01 ± 0.03	0.04
Number of maxillofacial cellulitis per month of dental follow‐up (*n*/month), mean ± SD	0.004 ± 0.02	0.007 ± 0.02	0.002 ± 0.008	0.23
Dental caries during dental follow‐up, *n* (%)	13 (18.3)	7 (18.4)	6 (18.2)	0.98

Twenty‐nine children (40.8%) were diagnosed with at least one abscess during the dental follow up while eight (11.3%) presented with a facial cellulitis of dental origin.

**Fig. 1:**
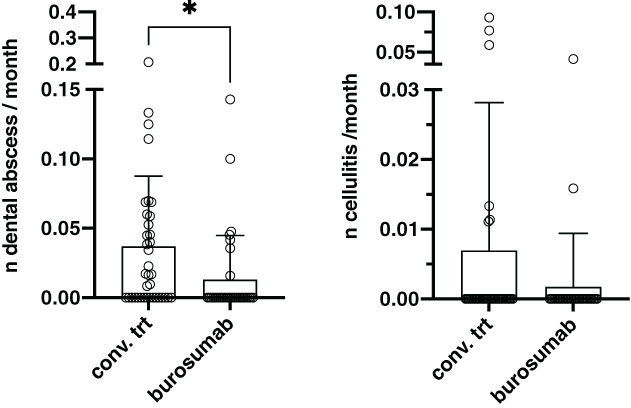
Comparison of the mean number of abscesses (*A*) and cellulites (*B*) per month of dental follow‐up in patients treated with conventional treatment or burosumab. Each point represents a child included in the study. Bars represent SD; *indicates *p* < 0.05.

The mean number of dental abscess per month of dental follow‐up was 0.03 ± 0.04 in the study population and was significantly decreased in the burosumab group when compared to the CT group (0.01 versus 0.04; *p* = 0.04) (Fig. 1A). The mean number of cellulitis was 0.004 ± 0.02 in the study population and was not significantly reduced in the burosumab group (0.002 versus 0.007; *p* = 0.23) (Fig. 1B). The percentage of patients with dental caries diagnosed during follow‐up was the same in the CT and burosumab groups (18%).

To assess the impact of age on the occurrence of abscesses, we compared the frequency of abscesses in the different age groups (<4 years; 4–8 years; 8–12 years; >12 years) irrespective of the treatment, in the CT group and in the burosumab group. We did not observe significant differences (Table S2) between age groups when all children were analyzed or in the CT group. In the burosumab group, the frequency of abscesses varied between the different age groups (*p* < 0.001), with more abscesses detected in the youngest group of children.

Results of the linear regression models are presented in Table 2. According to the regression models, in descending order of effect size, female gender, XLH treatment with burosumab, and age at XLH treatment onset were negatively and significantly associated with the number of dental abscess per month of dental follow‐up. Adjusted for sex and the age at XLH treatment onset, XLH treatment with burosumab was not significantly associated with the number of dental abscess per month. The female gender was the unique factor associated with fewer dental abscesses.

**Table 2:** Linear Regression Between Number of Dental Abscess/Month and Treatment Received (Burosumab or Conventional Treatment)

Variables	Beta coefficient	Univariate *p* value	Multivariate *p* value
Sex (female)	−0.04	0.0001	0.0002
Type of XLH treatment (burosumab)	−0.02	0.02	0.12
Age at XLH treatment onset	−0.0002	0.02	0.47
Dental follow‐up duration	−0.0003	0.09	
Age at dental follow‐up onset	−0.0002	0.15	
Alkaline phosphatase level	0.0004	0.3	
Dental caries during dental follow‐up	−0.009	0.49	
XLH treatment duration	0.0001	0.87	

We next analyzed separately the 19 children that had at least 1 year of dental follow‐up on both treatments in a matched data analysis (paired fashion) (Table 3). The mean age at XLH treatment onset and the mean age at dental follow‐up onset were higher in the burosumab group compared to the CT group, whereas the mean duration of XLH treatment and duration of dental follow‐up were lower. The mean ALP level during dental follow‐up was lower in the burosumab group compared to the CT group (287.8 versus 451 IU/L; *p* < 0.0001). The mean number of dental abscess per month of dental follow‐up was significantly decreased with burosumab when compared to CT (0.007 versus 0.08; *p* = 0.01). The mean number of cellulitis was unchanged 0.002 versus 0.005 (*p* = 0.43).

**Table 3:** Comparison of Selected Participant Characteristics in Patients Who Had Conventional Treatment and then Burosumab Treatment

Variables	Conventional treatment (*n* = 19)	Burosumab treatment (*n* = 19)	*p*
Sex, *n* (%)			
Male	7 (36.9)	7 (36.9)	
Female	12 (63.1)	12 (63.1)	1
Age at XLH treatment onset (years), mean ± SD	2.10 ± 2.07	9.75 ± 2.92	<0.0001
XLH treatment duration (months), mean ± SD	90.8 ± 36.3	35.0 ± 7.1	<0.0001
Alkaline phosphatase level (IU/L), mean ± SD	451.0 ± 147.2	297.8 ± 100.2	<0.0001
Age at dental follow‐up onset (years), mean ± SD	6.60 ± 3.67	10.36 ± 3.11	<0.0001
Dental follow‐up duration (months), mean ± SD	37.2 ± 27.8	27.7 ± 8.1	0.19
Number of dental abscess per month of dental follow‐up (*n*/month), mean ± SD	0.08 ± 0.1	0.007 ± 0.02	0.01
Number of maxillofacial cellulitis per month of dental follow‐up (*n*/month), mean ± SD	0.005 ± 0.02	0.002 ± 0.009	0.43
Dental caries during dental follow‐up, *n* (%)	2 (10.5)	4 (21.0)	0.68

**Fig. 2:**
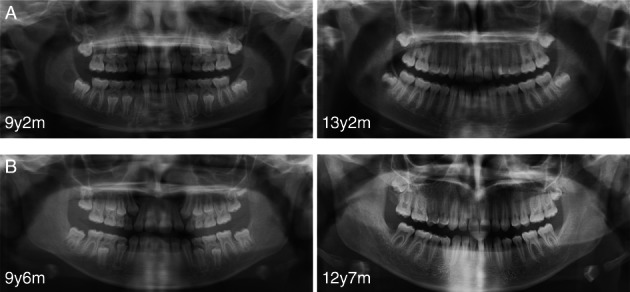
Dental orthopantomograms of two girls with XLH treated with conventional treatment (*A*) and burosumab (*B*). Note the enlarged pulp chambers, especially those of the first molars, and the improvement over time in both cases.

Finally, we compared the evolution of the size of the pulp chambers on successive radiographs, but we could not conclude on differences between treatments (Figs. 2A,B). Two successive orthopantomograms were not available for all patients, the time between the two radiographs varied, and the superposition of the two radiographs was not always possible. We observed in the two treatment groups great variations between children: no change over time, detectable but limited change, significant improvement (Fig. 2).

## Discussion

The structure of the tooth can be dramatically altered in patients with genetic skeletal disorders that disturb either mineral homeostasis or the extracellular matrix.^(^ ^14^, ^25^ ^)^ Such is the case of XLH, in which spontaneous dental abscesses are a direct consequence of constitutional defects of dentin. These abscesses particularly affect children, but also adults. They are responsible for premature tooth loss^(^ ^5^, ^18^ ^)^ with functional, aesthetic and social consequences^(^ ^11^, ^12^ ^)^ and may evolve into facial cellulitis. Therefore, one of the goals of XLH treatment is to reduce endodontic infections.^(^ ^1^ ^)^ The so‐called conventional treatment (CT) has shown its interest in reducing abscesses, both in children^(^ ^19^ ^)^ and in adults.^(^ ^18^, ^20^ ^)^ Phosphorus and vitamin D analogue supplementation improves dentin mineralization by reducing the proportion of unmineralized interglobular dentin and the presence of mineralization‐inhibiting peptides.^(^ ^26^, ^27^, ^28^ ^)^ More recently, several studies have shown the value of burosumab in the treatment of musculoskeletal manifestations of XLH in children.^(^ ^21^, ^23^ ^)^ The impact of burosumab on the mineralization of dental tissues and on oral complications is unknown. Here, for the first time, we report a possible beneficial effect of burosumab on the occurrence of endodontic infections (dental abscesses) in children.

In the study population, the incidence of a dental abscess during the dental follow‐up was very high, with 40.8% of the children presenting with at least one abscess, comparable with other studies.^(^ ^8^, ^10^ ^)^. Although most studies reporting dental abscesses in XLH are based on data collected during interviews with patients, we chose to focus on clinical data of events diagnosed during follow‐up by dentists experienced with XLH oral manifestations. We selected patients with at least 1 year of dental follow‐up in our center. The number of children included in our study constitutes the largest cohort of XLH children with dental follow‐up to the best of our knowledge.

It is acknowledged that defective dentin formation and mineralization during XLH are the main causes of spontaneous endodontic infections: abnormal dentin generates microcracks in the enamel that allow bacterial leakage, oral bacteria easily reach the enlarged pulp chambers, and the bacteria induce inflammation and then pulp necrosis.^(^ ^8^, ^17^ ^)^ The decrease in infections with burosumab would therefore imply an improvement of dentin formation and mineralization. In *Hyp* mice, a murine model that replicates the XLH phenotype, early treatment with an FGF23‐neutralizing antibody was shown to increase predentin formation and mineralization.^(^ ^24^ ^)^ It should be also noted that individuals with FG23 deficiency have increased dentin formation and reduced pulp chamber volume.^(^ ^29^ ^)^ Several biological mechanisms may contribute to the improvement of dentin mineralization with burosumab. Normalization of phosphatemia, via the reduction of excess FGF23 activity on the kidney and parathyroid gland, likely contributes to the improvement of mineralization, as it is observed with CT.^(^ ^17^ ^)^ Local targeting of excessive FGF23 in the environment of the mineralizing dentin by burosumab is also possible. In bone, increased FGF23 secretion was shown to directly contribute to mineralization defects, independent of phosphatemia.^(^ ^30^ ^)^ Whether such a mechanism exists in dentin remains unknown.

Another means of action of burosumab in reducing dental infections, independent of dentin, could be a modulation of the immune response. In chronic kidney disease, another condition with elevated circulating FGF23, high FGF23 serum level associates with increased risk of infection.^(^ ^31^ ^)^ It has been observed that high level of FGF23 can modulate neutrophils and other leukocytes’ function and recruitment and impair the immune response.^(^ ^32^, ^33^ ^)^ The elevated FGF23 in XLH could contribute in this way to the development of an infection and its rapid diffusion into soft tissue to evolve into cellulitis. Burosumab, by targeting the excessive FGF23 in XLH, may improve the immune response to endodontic infection and reduce the occurrence of dental abscess.

Treatment with burosumab was not associated with a significant reduction in cellulitis in our study. This may be related to the low number of events observed during follow‐up. We also observed no difference in the occurrence of dental caries between the groups.

A limitation of our study could be the difference in age at treatment initiation between the CT and burosumab groups, because the age at which treatment with burosumab is started likely affects the occurrence of abscesses.^(^ ^34^ ^)^ In their study, Ward and colleagues^(^ ^34^ ^)^ observed that in children under 5 years of age treated with burosumab, dental abscesses were absent, whereas they occurred in more than one‐half of older children. We could not confirm this finding, because we observed more abscess in the youngest children in the burosumab group. Of importance, the number of young children treated with burosumab is limited in our study, this being related to the relatively recent approval of burosumab and one of its indications; ie, the treatment of children who do not respond well to CT.

The age at which XLH treatments are started is an important factor influencing treatment outcomes on skeletal events.^(^ ^1^, ^16^ ^)^ Although this has not been formally demonstrated for dental involvement, it is very likely to be the case as well. Indeed, dental tissues including dentin are normally not remodeled and the potential for repair is very limited. Except for third molars, the formation and mineralization of the crown dentin takes place for the most part between 0.5 and 6.5 years of age for permanent teeth.^(^ ^35^ ^)^ It is expected that later treatment will have less effect on dental abscesses, and it is striking to observe that despite a later age of treatment in the burosumab group compared to the CT group, the incidence of abscesses was lower with burosumab. We also believe that it is the higher mean age of XLH treatment onset in the burosumab group that explains why age of treatment onset was associated with a reduction in the number of abscess in the regression linear model.

How burosumab could improve tooth structure in children after 7 or 8 years of age, is unknown. Dentin formation and mineralization continues during the life of the tooth at a much‐reduced rate,^(^ ^36^ ^)^ but it is well established that dentinogenesis can be induced with different stimuli, such as caries and trauma.^(^ ^37^ ^)^ It is possible that normalization of phosphatemia and/or targeting of FGF23 locally lift an inhibition of mineralization even after the crown formation of the tooth is complete. Indeed, in *Hyp* mice, FGF23‐neutralizing antibody improved the dentin crown volume by inducing the formation of reparative dentin.^(^ ^24^ ^)^

It is possible that the mean age of the group affects the frequency of abscesses, independently of the treatment, if abscesses were to occur preferentially at certain ages. To test this hypothesis, we measured the frequency of abscesses in the different age groups in children treated with CT. We did not observe significant differences (Table S2) and therefore consider that the mean age of the two groups does not explain the difference in abscess number observed between CT and burosumab.

The indication for the use of burosumab in case of failure of CT allows the comparison of both treatments in the same child. All children reported here were treated with CT before being treated with burosumab. Efficacy of burosumab treatment was shown by the decreased ALP level while under burosumab treatment. Comparison of the incidence of infections in the same patient according to treatment showed a clear advantage of burosumab. A possible bias in this comparison is the selection of XLH children who did not respond well to CT (with, among other things, more dental abscesses) and in whom the switch to burosumab therapy was proposed for this reason.

Finally, we observed a significantly reduced incidence of abscesses in girls compared to boys. It is unclear if this has been reported in the past and it is possible that this went unnoticed because of the higher incidence of XLH in females. An explanation could be the presence of a second nonmutated *PHEX* allele in females that may partially compensate for the mutated allele.

In conclusion, we report a reduced number of dental abscesses in XLH children treated with burosumab compared to XLH children treated with CT. Although these results are encouraging, they show that dental follow‐up is particularly important regardless of the treatment of XLH. They must be confirmed in other centers and in controlled prospective studies.

## Author Contributions

**Margaux Gadion:** Data curation; formal analysis; investigation. **Agathe Hervé:** Data curation; formal analysis; investigation. **Julia Herrou:** Conceptualization; data curation; formal analysis; writing – review and editing. Data curation; investigation; writing – review and editing. **Violaine Smail‐Faugeron:** Data curation; investigation. **Frédéric Courson:** Data curation; investigation; writing – review and editing. **Agnès Linglart:** Data curation; investigation; supervision; writing – review and editing. **catherine Chaussain:** Conceptualization; data curation; investigation; supervision; writing – review and editing.

## Conflicts of interest

The authors declared the following potential conflicts of interest with respect to the research, authorship, and/or publication of this article: AL and MBD have received honoraria, grant for research in other projects independent of this study from Kyowa Kirin Pharma. CC has received grants for research in other projects independent of this study from Kyowa Kirin Pharma. AR and VSF have received honoraria from Kyowa Kirin Pharma. All other authors have no relevant financial or nonfinancial interests to disclose.

### Peer Review

The peer review history for this article is available at https://publons.com/publon/10.1002/jbm4.10672.

## Supporting information


Table S1
Table S2
