# Supplementary material for: Burosumab and Dental Abscesses in Children With X‐Linked Hypophosphatemia
Source: JBMR Plus. 2022 Sep 20;6(11):e10672. doi: 10.1002/jbm4.10672 (PMC9664523; doi:10.1002/jbm4.10672)
Supplement: Supplementary file 1 — Table S1 Table S2 [file JBM4-6-e10672-s001.docx]

Appendix table 1: Comparison of selected participant characteristics of patients with at least one year of dental follow up while on conventional treatment and patients with at least one year of dental follow up on conventional therapy that were later switched to burosumab. Table values are mean ± SD unless otherwise stated.

| **Variables** | **TC only (n=38)** | **TC before burosumab switch (n=19)** | **Comparison between groups**  **(p value)** |
| --- | --- | --- | --- |
| Sex, n (%)  Male  Female | 18 (42.1)  22 (57.9) | 7 (36.9)  12 (63.1) | 0.71 |
| Age at XLH treatment onset, year | 2.92 (2.98) | 2.11 (2.07) | 0.31 |
| Alkaline phosphatase level, IU/L | 385.8 (147.7) | 451 (147.2) | 0.18 |
| Age at dental follow-up onset, year | 6.57 (3.33) | 6.61 (3.67) | 0.97 |
| Number of dental abscess per month of dental follow-up, n/month | 0.04 (0.05) | 0.08 (0.11) | 0.25 |
| Number of maxillo-facial cellulitis per month of dental follow-up, n/month | 0.007 (0.02) | 0.005 (0.02) | 0.97 |
| Dental caries during dental follow-up, n (%) | 7 (18.4) | 2 (10.5) | 0.70 |

Appendix table 2. Number of dental abscess and cellulitis occurring under conventional treatment by age category. Table values are mean ± SD. Number of patients in each group in [].

| **Age category** | **< 4 years** | **4-8 years** | **8-12 years** | **12-16 years** | **Comparison between groups**  **(P value)** |
| --- | --- | --- | --- | --- | --- |
| Number of dental abscess per month of dental follow-up, n/month | 0.05 (0.06)  [n=11] | 0.03 (0.05)  [n=25] | 0.02 (0.04)  [n=22] | 0.01 (0.02)  [n=13] | 0.27 |
| Number of dental abscess per month of dental follow-up, under conventional treatment, n/month | 0.03 (0.05)  [n=8] | 0.04 (0.06)  [n=18] | 0.04 (0.06)  [n=9] | 0.04 (0.03)  [n=3] | 0.99 |
| Number of dental abscess per month of dental follow-up, under burosumab, n/month | 0.08 (0.07)  [n=3] | 0.002 (0.006)  [n=7] | 0.007 (0.02)  [n=13] | 0.008 (0.02)  [n=10] | 0.0003 |
